# An Unusual Cause of Pulmonary Nodules in the Emergency Department

**DOI:** 10.1155/2015/278020

**Published:** 2015-02-23

**Authors:** Ryan Yu, Melanie Ferri

**Affiliations:** ^1^Department of Pathology and Molecular Medicine, McMaster University, Hamilton, ON, Canada L8S 4L8; ^2^Department of Diagnostic Imaging, Juravinski Hospital and Cancer Centre and McMaster University, Hamilton, ON, Canada L8V 5C2

## Abstract

We report a 51-year-old woman who presented to the emergency department with left-sided pleuritic chest pain 2 weeks after subtotal hysterectomy and bilateral salpingo-oophorectomy for a leiomyomatous uterus. Computed tomography scan of the chest revealed bilateral pulmonary nodules. Biopsy showed cytologically bland spindle cells without overt malignant features. Immunohistochemistry confirmed smooth muscle phenotype, in keeping with a clinicopathologic diagnosis of benign metastasizing leiomyoma (BML). BML does not frequently come to the attention of the emergency physician because it is rare and usually asymptomatic. When symptomatic, its clinical presentation depends on the site(s) of metastasis, number, and size of the smooth muscle tumors. Emergent presentations of BML are reviewed.

## 1. Introduction

Benign metastasizing leiomyoma (BML) is an entity in which benign-appearing uterine smooth muscle tumors are associated with similar-appearing tumors at distant sites [1]. The lung is the most common site of involvement and usually shows multiple, occasionally solitary, well-circumscribed nodules, ranging in diameter from a few millimeters to several centimeters [2]. The finding of multiple pulmonary nodules raises a broad differential diagnosis, including primary or secondary neoplasms, vasculitis, collagen vascular disease, and granulomatous diseases. BML does not frequently come to the attention of the emergency physician because it is rare and usually asymptomatic. However, BML may exhibit a range of clinical presentations, some emergent, depending on the site of involvement, number, and size of the smooth muscle tumors (leiomyomas). We report a patient with benign metastasizing leiomyoma who presented in the emergency department with pleuritic chest pain.

## 2. Case Report

**Figure 1:**
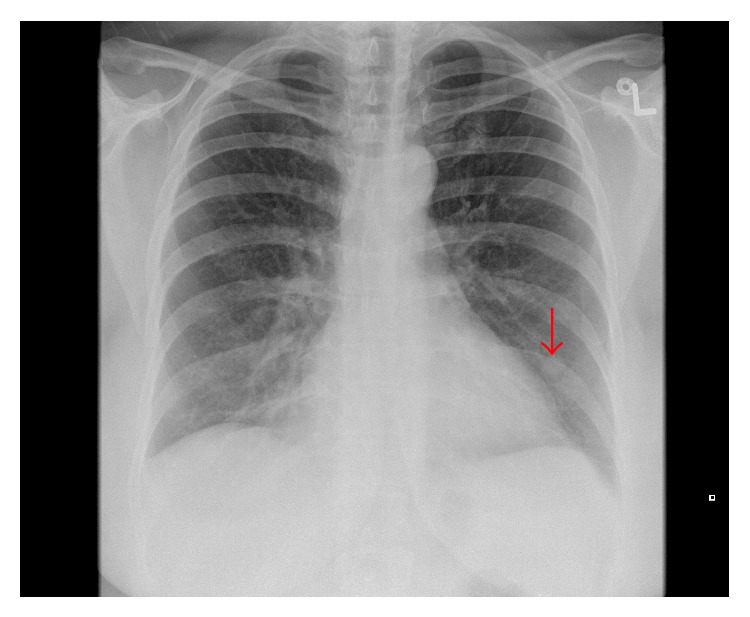
PA chest radiograph: there is a 1.3 cm nodule within the left lower lobe (arrow), projected lateral to the left cardiac border.

**Figure 2:**
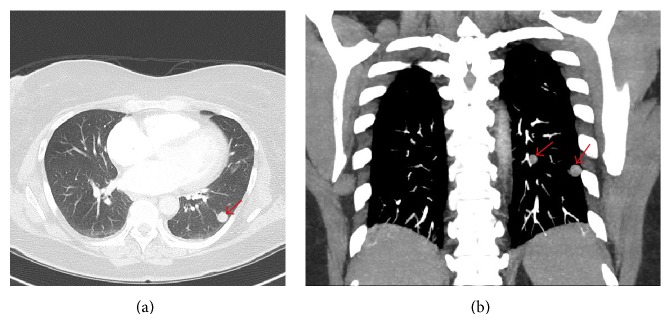
CT pulmonary angiogram performed the same day as the chest radiograph. (a) Axial image (lung windows): left lower lobe soft tissue nodule corresponding to the abnormality on the CXR (arrow) demonstrates no internal calcification or cavitation. Six other similar-appearing nodules of varied sizes were scattered throughout the lungs. (b) Coronal MIP image (soft tissue windows): two well-circumscribed left lower lobe nodules (arrows).

A 51-year-old woman, gravida 2 para 2, presented to the emergency department with a 2-day history of left-sided pleuritic chest pain. Two weeks prior, she underwent subtotal hysterectomy and bilateral salpingo-oophorectomy for a leiomyomatous uterus which was approximately the size of a 12-week gravid uterus. Ten years prior, she underwent a hysteroscopic myomectomy for a submucous leiomyoma. Her medical history was further remarkable for endometriosis, primary biliary cirrhosis, chronic cholecystitis, hypertension, hypercholesterolemia, and transient ischemic attack. On physical examination in the emergency department, she was afebrile with a blood pressure of 150/87, heart rate 60/min, respiratory rate 18/min, and oxygen saturation 99% on room air. She had a BMI of 33, normal heart sounds, and clear chest on auscultation. ECG was normal. ABG showed pH 7.41 and pCO_2_ 39 mmHg. She had a normal complete blood count, basic metabolic panel, and troponin. D-dimer was 1.2 *μ*g/mL FEU (reference: less than 0.5 *μ*g/mL FEU). Chest radiograph showed a 1.3 cm nodule in the left lower lobe (Figure 1) compared with a chest radiograph performed 4 years earlier which was clear. CT pulmonary angiogram (CTPA) showed bilateral, well-circumscribed, noncalcified, and noncavitated pulmonary nodules (Figures 2(a) and 2(b)) concerning for metastatic deposits. The nodules were not present on a chest CT performed 8 years earlier for the same indication. She was referred for thoracic surgery consultation.

**Figure 3:**
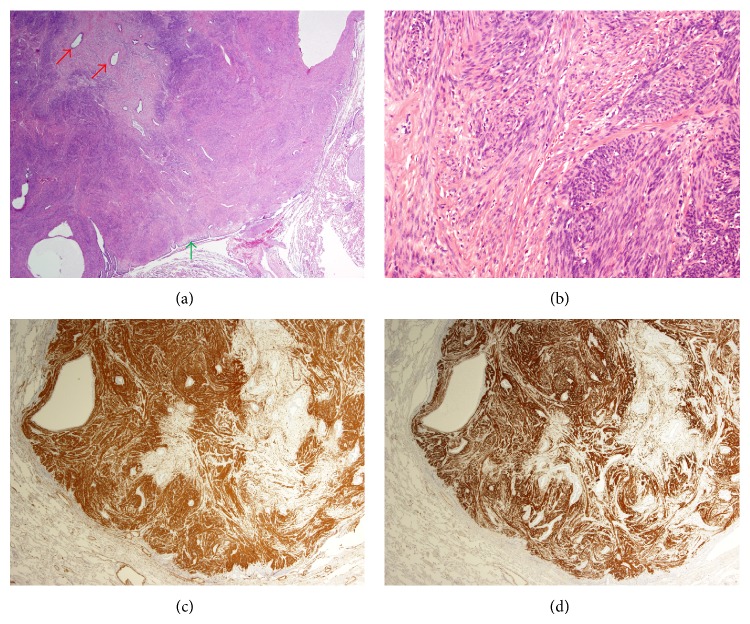
(a) A well-circumscribed tumor with a pushing border to the lung parenchyma (green arrow) (H&E, 40x). Note entrapped bronchiolar epithelium encircled by collagen (red arrows). (b) Bland smooth muscle cells without cytological atypia (H&E, 100x). (c) Diffuse staining (brown) for SMA (12.5x). Note negative staining (white) of collagen and bronchiolar epithelium in the tumor. (d) Diffuse staining (brown) for desmin (12.5x). There was negative tumor cell staining for p16, p53, WT-1, CD10, CD31, HMB-45, CD117/c-kit, and ALK-1 (not shown).

Subsequent mammogram and CT scan of the abdomen, pelvis, and head showed no other deposits or suggestion of a primary malignancy. She was taken to the operating room for diagnostic wedge resection of one of the nodules by VATS and a hilar lymph node biopsy. She tolerated the procedure well and was discharged from hospital on the third postoperative day without any complications. Microscopic examination of the resected nodule showed a a well-circumscribed, nonencapsulated tumor with a smooth pushing border to the surrounding lung parenchyma (Figure 3(a)). The tumor was composed predominantly of intersecting fascicles of bland smooth muscle cells without cytological atypia (Figure 3(b)). There was no necrosis and less than 1 mitotic figure per 10 high-power fields. On immunohistochemistry, the tumor cells showed strong, diffuse staining for *α*-smooth muscle actin (*α*-SMA) (Figure 3(c)), desmin (Figure 3(d)), and estrogen receptor. There was weak, diffuse staining for progesterone receptor. The Ki67 proliferation index was less than 1%. The findings were consistent with leiomyoma. The hilar lymph node showed reactive changes. In the context of bilateral pulmonary nodules and previously diagnosed uterine smooth muscle tumors, the clinical diagnosis was in keeping with benign metastasizing leiomyoma. Because the patient had a bilateral salpingo-oophorectomy, further growth of the nodules was not anticipated and she opted for periodic monitoring. She was well at 8-month follow-up from presentation in the emergency department, at which time repeat chest CT showed no interval change in the number or size of the pulmonary nodules.

## 3. Discussion

BML is rare, despite the high incidence of uterine leiomyomas in the general population. It occurs in women at an average age of 47 years, most of whom have undergone hysterectomy or myomectomy for uterine leiomyomas [3]. The clinical presentation of BML depends largely on its site of involvement, of which the lung is most frequent. BML involving the lung is usually asymptomatic and found incidentally on imaging, such as during staging workup for unrelated malignancies [4]. The nodules are usually found after hysterectomy or after myomectomy at durations of 3 months to 20 years [3]. BML may also be found concurrently at diagnosis of uterine leiomyomas in patients who present with leiomyoma symptoms (i.e., vaginal bleeding and abdominal or pelvic pain) [5] or in the perioperative period [6]. Uncommonly, BML involving the lung presents with a nonproductive cough or mild chest/back pain. Shortness of breath is also uncommon and tends to present late in patients when the number or size of the nodules begins to compromise lung function. Respiratory failure secondary to a large leiomyoma of the left lower lung that extended and obstructed the right mainstem bronchus has been reported [7]. Rarely, BML presents with hemoptysis [8], pneumothorax associated with lung cysts [5], hemothorax [9], or empyema [10]. Further, BML may have extrapulmonary manifestations including a systolic murmur by right ventricular metastasis [11]; abdominal pain by pelvic metastasis [12]; jaundice by metastasis to the pancreatic head [13]; arm and shoulder pain by metastatic compression of the infraclavicular brachial plexus [14]; severe low back pain and saddle anesthesia by metastasis to the S2 vertebral body with expansion into the spinal canal [15]; and abducens and hypoglossal nerve palsy by metastasis to the posterior cranial fossa [15].

BML lung nodules typically do not calcify or enhance after intravenous contrast administration [5]. They may cavitate [16] and raise additional diagnostic considerations on imaging. Pathologic examination of a BML nodule is necessary to establish the diagnosis, because it allows confirmation of a smooth muscle phenotype by immunohistochemistry (SMA-positive tumor cells). In the clinical context of a concurrent or previously diagnosed uterine smooth muscle tumor(s), a presumptive diagnosis of BML may be rendered. In the absence of history or evidence of a uterine smooth muscle tumor(s), the presumptive diagnosis is multiple fibroleiomyomatous hamartoma, which is histologically indistinguishable from BML. Definitive diagnosis requires molecular analysis of the uterine and pulmonary tumors [17], which is not performed in most cases.

The exact pathogenesis of BML is unknown. Three hypotheses have been proposed [17]: (1) BML represents proliferation of smooth muscle that is native to the lungs; (2) BML gains venous access from the trauma of myomectomy or hysterectomy; (3) BML represents a very low-grade leiomyosarcoma. The first two hypotheses are problematic because BML may involve extrapulmonary sites and may occur in the absence of uterine surgery, respectively. The third hypothesis is most widely accepted and likely reflects sampling error on grossing of the uterine smooth muscle tumor(s).

**Figure 4:**
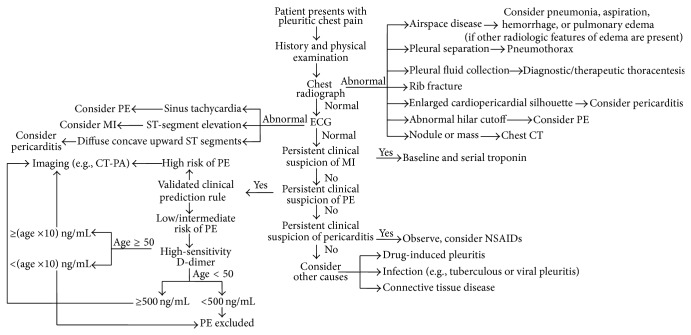
Diagnostic algorithm for pleuritic chest pain (modified from Kass et al. [18] and Cuker [21]).

Investigation and management in the emergency department must be tailored to the particular BML presentation. As mentioned, BML involving the lung is usually asymptomatic. Its presentation as pleuritic chest pain is uncommon and invokes a broad differential diagnosis [18], the most critical of which include pneumothorax, myocardial infarction, and pulmonary embolus (PE) (Figure 4). In this case, the patient's risk of PE was low/intermediate by Wells score [19]. Without evidence of PE on CTPA, her elevated D-dimer most likely reflects the normal process of recovery after hysterectomy. In current practice, a normal D-dimer (i.e., below a cut-off value of 500 *μ*g/L) may allow the exclusion of PE. However, alternative D-dimer cut-offs may exclude PE more reliably in clinical settings where D-dimer may be elevated for another reason(s), such as older patients [20, 21], postsurgery, and malignancy.

Suspected lung metastases of unknown primary should be referred for biopsy. There is no standardized management approach for lung involvement by BML. Because most lesions stay constant in size for a long time, a wait-and-see strategy consisting of periodic serial imaging is usually reasonable. This strategy also allows detection of lesions suspicious for lung adenocarcinoma, which may be present concurrently among the BML nodules [22] or develop in the course of BML monitoring [23]. Surgical resection of the BML nodules, if feasible, should be considered for prophylaxis of complications observed by some authors [24]. Given the presence of estrogen and progesterone receptors [25], BML is thought to be hormonally responsive. Surgical removal of estrogen stimulation may be accomplished with bilateral oophorectomy. Alternatively, for unresectable lung nodules or patients who prefer nonsurgical management, hormonal therapeutic options that include tamoxifen, progesterone, aromatase inhibitors, luteinizing hormone-releasing hormone analogue, and estrogen receptor modulators should be considered [26, 27].

In conclusion, emergency physicians should be aware of BML and its range of clinical presentations. Although BML involving the lung is usually asymptomatic and found incidentally, it may be present with emergent symptoms. BML should be considered in women of reproductive age with current or previously diagnosed uterine smooth muscle tumor(s) who are found to have multiple pulmonary nodules in the absence of pertinent history, risk factors, or localizing findings suggestive of metastasis from other primary site.

## Abbreviations

ABG: Arterial blood gas
ALK-1: Anaplastic lymphoma kinase-1
Bcl-2: B-cell lymphoma-2
BMI: Body mass index
BML: Benign metastasizing leiomyoma
CD: Cluster of differentiation
CT: Computed tomography
CTPA: Computed tomography pulmonary angiogram
CXR: Chest X-ray
ECG: Electrocardiogram
FEU: Fibrinogen equivalent units
H&E: Hematoxylin and eosin
HMB-45: Human melanoma black-45
MI: Myocardial infarction
MIP: Maximum intensity projection
NSAID: Nonsteroidal anti-inflammatory drug
PA: Posterior-anterior
PE: Pulmonary embolus
SMA: Smooth muscle actin
VATS: Video-assisted thoracic surgery
WT-1: Wilms tumor-1.
